# Inhalations of Oxygen

**Published:** 1885-02

**Authors:** 


*Inhalations of Oxygen.*—Massei agrees with Leci and Boucher in praising the efficacy of the inhalations of oxygen in diphtheria. This gas is a poison to bacteria and a stimulant in the adynamy and in the threatening paralysis of the vagus.—*La Sperimentale.*

